# Impact of antihypertensive treatment on cardiovascular event reduction in patients with asymptomatic carotid artery stenosis: a systematic review and meta-analysis

**DOI:** 10.11604/pamj.2025.52.18.46768

**Published:** 2025-09-16

**Authors:** Apurva Popat, Gauri Pethe, Sweta Yadav, Srinivasulu Sathipati, Param Sharma

**Affiliations:** 1 Department of Internal Medicine, Marshfield Clinic, Wisconsin, United States of America,; 2 Department of Rheumatology, Marshfield Clinic, Wisconsin, United States of America,; 3 Marshfield Clinic, Wisconsin, United States of America,; 4 Department of Cardiology, Marshfield Clinic, Wisconsin, United States of America

**Keywords:** Antihypertensive treatment, asymptomatic carotid artery stenosis, cardiovascular conditions, calcium channel blockers, beta-blockers

## Abstract

Cardiovascular conditions disrupt the normal functioning of the heart and blood vessels, often due to underlying conditions like atherosclerosis or hypertension. Antihypertensive medications are essential in cardiovascular disease management, encompassing several major drug classes with distinct mechanisms of action. Hence, this review evaluated the impact of various antihypertensive treatments on cardiovascular event reduction in asymptomatic carotid artery stenosis (CAS) patients. A comprehensive literature search was conducted from inception to 2024 on various databases by using specific keywords, and based on the eligibility criteria, three observational cohort studies and six randomized controlled trials (RCTs) of the 540 records retrieved were incorporated in this systematic review. The Newcastle-Ottawa scale was used to assess the methodological quality of the cohort studies, and the risk of bias visualization tool was used for RCTs. Data were then systematically extracted and analyzed. The results reported that enalapril and fosinopril demonstrated dual benefits in blood pressure (BP) reduction and vascular remodeling, though meta-analysis showed statistically insignificant improvements in regional cerebral blood flow (CI: -0.84, 6.08, P = 0.14, I^2^= 94%). Similarly, isradipine, lacidipine, and amlodipine improved carotid hemodynamics and cerebral perfusion, with meta-analysis favoring calcium channel blocker intervention for blood pressure management (CI: -3.25 to 7.64, P = 0.43). On the other hand, thiazide diuretics effectively reduced BP but showed limited efficacy in preventing atherosclerosis progression. In addition, angiotensin II receptor blockers (ARBs) significantly reduced 5-year stroke rates from 11% to 3.5%. Moreover, beta-blockers showed specific benefits, with metoprolol improving plaque echogenicity (57.3 ± 16.8 vs. 51.8 ± 20.0, p = 0.006) and reducing cardiovascular events (17% vs. 37% placebo, p = 0.011), while labetalol effectively managed post-endarterectomy hypertension. In conclusion, antihypertensive treatments showed varying effectiveness in cardiovascular event reduction and improvements in vessel measures.

## Introduction

Globally, cardiovascular disorders remain a leading cause of morbidity and mortality, with particular significance in patients with vascular disease [1]. These events encompass a spectrum of acute and chronic manifestations, including myocardial infarction, stroke, transient ischemic attacks, and cardiovascular death [2,3]. The prevention of such events has become increasingly important in modern medical practice, especially among those with pre-existing vascular conditions [4]. The duration and intensity of blood pressure management have emerged as critical factors in determining cardiovascular outcomes, and prompt early intervention may provide superior long-term benefits [5].

Asymptomatic carotid artery stenosis (CAS) is attributed to the narrowing of carotid arteries without overt neurological symptoms [6,7]. Despite its asymptomatic nature, it serves as a major risk factor for other cardiovascular events and stroke [8,9]. The pathological difference between asymptomatic and symptomatic CAS is mainly associated with stability and plaque morphology. Asymptomatic CAS demonstrates low inflammatory activity with a thicker fibrous cap showing more stable plaques and leading to a lower risk of immediate rupture, but consistent vascular burden for a long period. In contrast, symptomatic CAS involves more rupture-prone unstable plaques, demonstrating large lipid core, thin fibrous cap, and increased cell infiltration due to inflammation, which contributes to embolism and acute ischemic events [8-11]. CAS involves complex interactions between hemodynamic forces, inflammatory processes, and vascular remodeling influenced by different antihypertensive agents [10,11]. The association between CAS severity and cardiovascular risk is continuous, with greater degrees of stenosis associated with higher event rates [12].

Antihypertensive medications are essential in cardiovascular disease management, encompassing several major drug classes with distinct mechanisms of action [10,13,14]. Angiotensin receptor blockers (ARBs) and angiotensin-converting enzyme (ACE) inhibitors target the renin-angiotensin-aldosterone system, with agents such as telmisartan, valsartan, losartan, irbesartan, azilsartan, olmesartan, candesartan, and eprosartan demonstrating significant efficacy in blood pressure management [15,16]. Whereas, calcium channel blockers (CCBs), including nifedipine, felodipine, amlodipine, isradipine, diltiazem, clevidipine, nicardipine, and verapamil modulate the influx of calcium in vascular smooth muscle cells, causing vasodilation and reduced peripheral resistance [17]. In addition, beta-blockers consisting of metoprolol, carvedilol, atenolol, propranolol, nebivolol, bisoprolol, labetalol, esmolol, nadolol, timolol, pindolol, and sotalol, reduce cardiac workload through sympathetic nervous system inhibition [18]. Thiazide diuretics, including hydrochlorothiazide, chlorthalidone, indapamide, metolazone, and bendroflumethiazide, decrease blood volume through enhanced sodium excretion [19].

Hence, the current study is performed to comprehensively explore the impact of antihypertensive treatment in asymptomatic CAS patients for the reduction of cardiovascular events by assessing the effectiveness of various classes of antihypertensive medications in this population, with particular consideration of their effects on carotid plaque progression and stability, and to understand their impacts on different outcomes of cardiovascular disease, including myocardial infarction, stroke, and cardiovascular death.

## Methods

The current review adhered to the Preferred Reporting Items for Systematic Reviews and Meta-Analysis (PRISMA) guidelines [20].

**Identification and selection of studies:** a comprehensive literature search was conducted from inception to 2024 on the Web of Science, Cochrane Library, Cumulative Index for Nursing and Allied Health Literature (CINAHL), PubMed, and Medline databases.

**Search strategy:** the keywords used with medical subject headings (MeSH) terms in different combinations to optimize the search consisted of: “antihypertensive treatment”, or “angiotensin II receptor blockers”, or “irbesartan”, or “valsartan”, or “losartan”, or “candesartan”, or “eprosartan”, or “olmesartan”, or “azilsartan”, or “telmisartan”, or “calcium channel blocker”, or “amlodipine”, or “nifedipine”, or “felodipine”, or “nicardipine”, or “isradipine”, or “clevidipine”, or “verapamil”, or “diltiazem”, or “atenolol”, or “beta blockers”, or “metoprolol”, or “bisoprolol”, or “propranolol”, or “carvedilol”, or “nebivolol”, or “labetalol”, or “esmolol”, or “nadolol”, or “timolol”, or “pindolol”, or “sotalol”, or “thiazide diuretics”, or “hydrochlorothiazide”, or “chlorthalidone”, or “indapamide”, or “metolazone”, or “bendroflumethiazide”, and “carotid artery stenosis”, or “carotid artery constriction”, or “carotid artery narrowing”, or “carotid artery occlusion”, or “carotid artery obstruction”, or “reduced lumen of the carotid artery”, or “carotid atherosclerosis with stenosis”, or “chronic carotid artery disease”.

**Study selection:** for inclusion in the study, the articles were screened and selected by two reviewers independently. The conflicts among the reviewers were resolved collaboratively or through discussion with the third reviewer. The retrieved results were exported to Zotero screening software, which automatically detected retracted articles. In addition, it identified duplicate records, which a reviewer manually merged. The reviewers then screened the remaining articles.

**Eligibility criteria:** this systematic review included research related to the impact of antihypertensive therapy on cardiovascular event reduction in patients with asymptomatic CAS that was published from inception to 2024, described in the English language with full-text availability. The articles were selected based on the modified population, intervention, comparison, primary outcomes, and study design (PICOS) criteria [21]. The PICOS criteria in the present review were described as P: patients with asymptomatic CAS, I: antihypertensive therapy, C: various antihypertensive therapies, O: cardiovascular event reduction, and S: observational cohort studies or randomized controlled trials (RCTs). Whereas, the exclusion criteria consisted of articles not related to the context, non-availability of full-text, and not described in English. Moreover, letters, opinion pieces, editorials, conference abstracts, study protocols, reviews, and meta-analyses were also excluded.

**Methodological quality assessment:** for methodological quality assessment of the RCTs, the risk of bias visualization (robvis) tool was used [22,23]. Additionally, to evaluate the risk of bias (ROB) for cohort studies, the Newcastle-Ottawa Scale (NOS) was utilized [24].

**Data selection and extraction:** data sets consisting of authors, study settings, and designs, and population characteristics (size, age, intervention type, primary outcomes, secondary outcomes, objectives, and findings) were systematically extracted from the included studies and tabulated using Microsoft Excel 2019.

**Data analysis:** the extracted data were analyzed according to the focused outcome measures. For meta-analysis, Review Manager software version 5.4.1 (RevMan, Cochrane Collaboration) was used to pool quantitative data from included studies. Standardized mean differences (SMD) were calculated for continuous outcomes (e.g., rCBF for ACE inhibitors and BP for calcium channel blockers) with 95% confidence intervals (CI) to assess the effect of antihypertensive interventions. The SMD was chosen as the effect measure to account for variations in measurement scales across studies. A random-effects model was applied due to anticipated clinical and methodological heterogeneity, such as differences in patient populations, intervention durations, and study designs. Heterogeneity was assessed using the I^2^ statistic and chi-squared test, with I^2^values >50% indicating substantial heterogeneity, prompting exploration of potential sources. Forest plots were generated in RevMan to visualize pooled SMDs, CIs, and p-values, with the software´s ‘inverse variance´ method used for pooling continuous data.

Sensitivity analyses were conducted to evaluate the robustness of results by excluding studies with a high risk of bias or those with shorter follow-up periods, as identified during quality assessment. No subgroup analyses were performed due to the limited number of studies and insufficient data on patient subgroups (e.g., by age, sex, or stenosis severity), though such analyses are recommended for future research. Publication bias was not formally assessed due to the small number of studies included (fewer than 10), as per Cochrane recommendations, but funnel plots were visually inspected in RevMan where feasible to detect potential reporting bias. Missing data were addressed by excluding studies with incomplete outcome reporting from the meta-analysis. All statistical tests were two-sided, with a significance threshold of p < 0.05.

## Results

**Figure 1:**
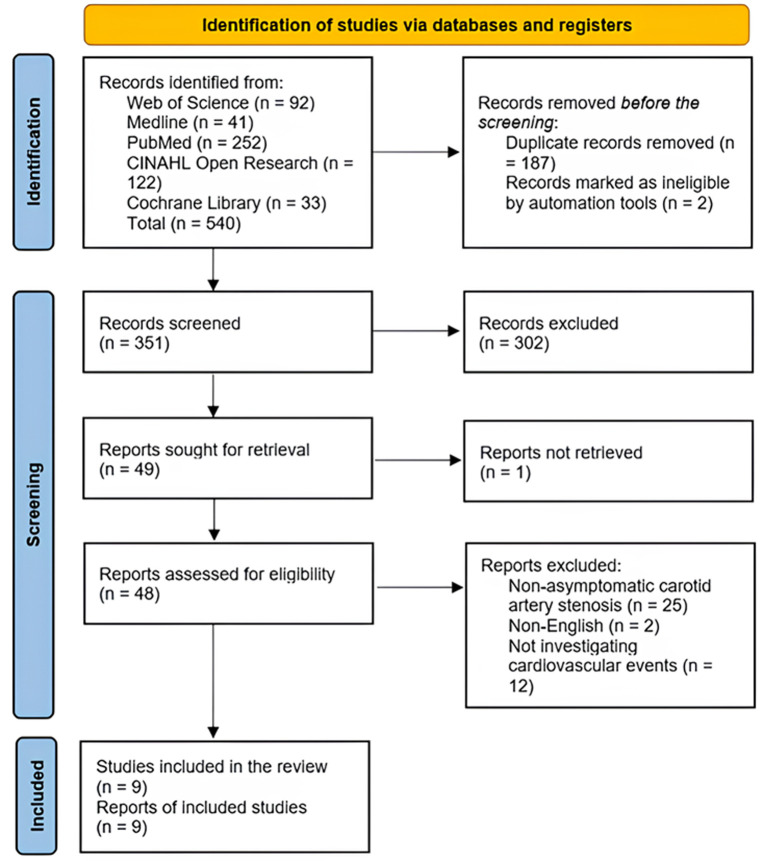
study selection strategy

**Study selection:** initially, from the overall 540 records, 187 duplicate records were eliminated. Additionally, two ineligible records were retracted by the automation tools. Furthermore, 302 records were excluded following the screening of the titles and abstracts of the studies. Of the remaining 49 articles identified for retrieval, one study could not be accessed. Following this, for eligibility, 48 articles were assessed, after which 39 articles were excluded due to patients with non-asymptomatic CAS, non-availability of articles in the English language, and articles not investigating cardiovascular events. Finally, three cohort studies and six RCTs that fulfilled the criteria were incorporated [25-33]. The selection strategy for studies according to the PRISMA flow diagram is illustrated below (Figure 1).

**Figure 2:**
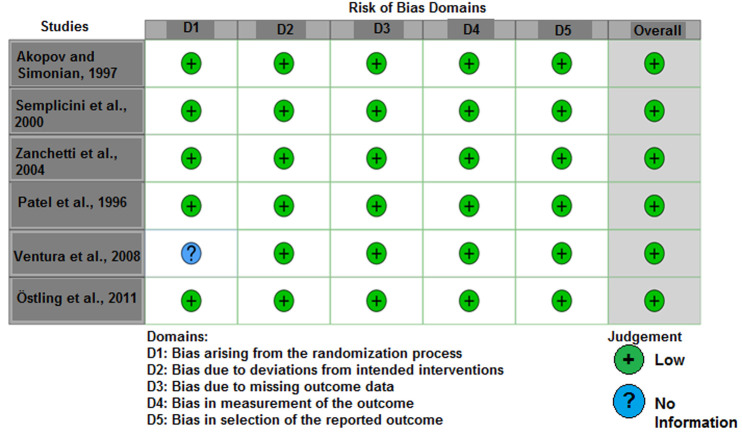
traffic lights plot the Robvis assessment results

**Methodological quality assessment:** the cohort studies showed a favorable methodological quality (Annex 1). In addition, the included RCTs demonstrated an overall low ROB (Figure 2).

**Data selection and extraction:** this review included RCTs and observational studies investigating the effects of antihypertensive medications on carotid vascular health across different regions, including Armenia, Denmark, Spain, the USA, Sweden, and Italy. Moreover, the study population included patients with asymptomatic CAS. The studies explored various antihypertensive medications that involved ARBs, CCBs, beta-blockers, and specific medications like isradipine, enalapril, valsartan, and candesartan (Table 1). In addition, the studies examined outcomes including carotid vascular resistance, intima-media thickness, blood pressure management, carotid plaque progression, and cerebrovascular effects (Table 2, Table 3).

**Table 1:** demographic and population characteristics of the studies reporting overall condition and intervention types

Authors	Year	Country	Study design	Population characteristics i.e. size, age	Overall Condition	Interventions type
Fagan *et al*.	1991	United States of America	Observational cohort study	2 patients	Asymptomatic internal carotid artery	Prazosin hydrochloride, enalapril maleate
Jørgensen and Schroeder	1993	Denmark	Observational cohort study	95 patients	High-grade carotid artery stenosis (CAS)	labetalol
Chang *et al*.	2013	United States of America	Retrospective cohort study	7255 patients	Asymptomatic CAS	Statins, clopidogrel, ACE inhibitors/ARBs, and other standard therapies
Akopov and Simonian	1997	Armenia	Randomized controlled trial (RCT)	73 patients with essential hypertension	Asymptomatic	Isradipine, enalapril
Semplicini *et al*.	2000	Italy	RCT	15 hypertensive patients	Asymptomatic essential hypertension with moderate carotid atherosclerosis	Lacidipine, Hydrochlorothiazide (HCTZ)
Zanchetti *et al*.	2004	Italy	RCT	508 patients with asymptomatic carotid atherosclerosis	Asymptomatic carotid atherosclerosis	Group A: HCTZ, Group B: Fosinopril, Group C: HCTZ + Pravastatin, and Group D: Fosinopril + Pravastatin
Patel *et al*.	1996	United States of America	RCT	15 patients aged 60-79 years (6 women, and 9 men)	Asymptomatic hypertensive patients with ≥70% CAS	ramipril, enalapril
Ventura *et al*.	2008	Spain	RCT	26 patients	Carotid atherosclerosis	Atorvastatin (ATV), Atorvastatin + Amlodipine (ATV+AML)
Östling *et al*.	2011	Sweden	RCT	341 participants	Asymptomatic carotid artery disease	Metoprolol

**Table 2:** characteristics of the included observational studies reporting the impact of antihypertensive treatment in asymptomatic carotid artery stenosis

Authors	Year	Country	Objectives	Primary outcomes	Secondary outcomes	Findings
Fagan *et al*.	1991	United States of America	To assess the effects of antihypertensive medication on CBF and asymmetry in patients with carotid artery occlusion	Changes in cerebral blood flow (CBF) asymmetry and mean CBF after intervention	Regional flow changes, interhemispheric flow differences, and response patterns in the affected hemispheres	Prazosin improved mean CBF in the affected hemisphere, and reduced interhemispheric asymmetry, and enalapril decreased mean CBF bilaterally
Jørgensen and Schroeder	1993	Denmark	To investigate cerebral hyperperfusion after carotid endarterectomy and its relationship with defective autoregulation using transcranial Doppler	Postoperative ipsilateral headache, cerebral hyperperfusion symptoms, and seizures	Ipsilateral Vmean changes; reduced CO_2_ reactivity; internal carotid artery (ICA) stump pressure; symptom duration (short-term or prolonged)	18 patients experienced cerebral hyperperfusion symptoms. Symptoms ended with arterial pressure reduction using labetalol. Pressure difference across the stenosis was significantly higher in symptomatic patients
Chang *et al*.	2013	United States of America	To evaluate the effect of contemporary medical management on stroke prevention in asymptomatic CAS	Stroke rate among asymptomatic CAS patients	Impact of statin use on stroke risk	Statin use was associated with a significantly lower stroke rate (1.6% vs. 3.9%). Medical management reduced stroke incidence compared to older data

**Table 3:** characteristics of the included randomized controlled trials reporting the impact of antihypertensive treatment in asymptomatic carotid artery stenosis

Authors	Year	Country	Objectives	Primary outcomes	Secondary outcomes	Findings
Akopov and Simonian	1997	Armenia	To detect cerebrovascular effects of enalapril and isradipine in moderate hypertension patients	Effects on carotid vascular resistance and regional CBF	Side-to-side asymmetry in CBF depending on ICA stenosis severity	Isradipine was found to be beneficial (variant II) in patients with moderate or no stenosis of ICA, but caused a reduction in cerebral perfusion in 43.5% of patients with severe stenosis of ICA. Whereas enalapril was safe (variant I) for most patients, with only 13% experiencing reduced cerebral perfusion in severe stenosis of ICA
Semplicini *et al*.	2000	Italy	To assess the impact of HCTZ and lacidipine on regional cerebral perfusion in hypertensive asymptomatic patients with CAS	HCTZ increased mean relative perfusion (MRP) only in cortical areas. However, lacidipine elevated MRP in both cortical and subcortical regions	The mean change in local vascular resistance was -12 A.U. for HCTZ (p < 0.001) and -20 A.U. for lacidipine	Lacidipine improved both cortical and subcortical perfusion, while HCTZ improved only cortical perfusion
Zanchetti *et al*.	2004	Italy	To test whether fosinopril is more effective than HCTZ in slowing carotid atherosclerosis progression, in comparison to placebo, is pravastatin more effective when added to treatment, and if additive effects occur between ACE inhibition and lipid-lowering therapies	Progression of carotid atherosclerosis (CBMmax). Group A showed significant intima-media thickness (IMT) progression. Groups B, C, and D had no significant IMT progression, differing significantly from group A	Changes in distal common carotid IMT (CC-IMT) and carotid bifurcation IMT (Bif-IMT). Clinic and ambulatory BP reductions. Decrease in total and low-density lipoprotein cholesterol in groups C and D	HCTZ alone caused IMT progression. Fosinopril and pravastatin (alone or combined) prevented IMT progression. Additive effects of fosinopril and pravastatin were not formally significant but beneficial
Patel *et al*.	1996	United States of America	To determine changes in rCBF caused by enalapril and ramipril, and assess the impact of age on drug-induced alterations in rCBF	Change in regional CBF (rCBF)	Influence of age on rCBF changes, assessment of the lateralization of blood flow	No significant change in rCBF with ramipril, enalapril, or placebo. Ramipril and enalapril are safe starting doses in patients with ≥70% CAS
Ventura *et al*.	2008	Spain	To examine the impact of combined ATV and AML therapy on plaque inflammation and blood in hypertensive patients with carotid atherosclerosis	Reduction in inflammatory markers in blood	Lipid profile improvement: reduction in low-density lipoprotein and total cholesterol levels	Combined ATV and AML treatment was more effective in reducing inflammatory markers and macrophage infiltration compared to atorvastatin alone
Östling *et al*.	2011	Sweden	To examine whether the decrease in IMT progression rate in the carotid bulb induced by metoprolol CR/XL was accompanied by changes in carotid plaque echogenicity	Change in plaque	Frequency of plaques becoming more echo lucent; correlation of Gray scale median (GSM) changes with IMT progression rate	Plaques were more echogenic in metoprolol-treated participants. Greater increase in GSM from baseline in the metoprolol group

**Meta-analysis inhibitor treatment outcomes of Angiotensin-converting enzyme (ACE):** ACE inhibitors are essential in decreasing the incidence of cardiovascular events in asymptomatic CAS. Enalapril improved the carotid hemodynamic profile without inducing any significant harmful effect on cerebral blood flow (CBF) [28]. In addition, enalapril consistently lowered systemic BP with enhancement of regional carotid circulation. However, inter-individual variability in carotid remodeling responses ranged from minimal disease regression to significant progression.

Additionally, fosinopril lowered BP and retarded the carotid atherosclerosis process, an important marker for stroke risk [30]. There was significantly less CC-IMT progression in patients treated with fosinopril compared to hydrochlorothiazide (HCTZ)-treated subjects, demonstrating the dual action of ACE inhibitors in managing both the hemodynamic and structural components of carotid artery disease. Similarly, administration of enalapril led to a measurable decline in systolic and diastolic BP [25]. In one patient with left internal carotid artery occlusion (LICO), a decrease in BP from 140/79 mm Hg to 128/78 mm Hg was observed after enalapril administration, reflecting the efficacy of ACE inhibitors for hypertension management.

Enalapril and ramipril intervention was found to be particularly safe and effective in the case of CAS. Patel *et al*. (1996) found that a single dose of 5 mg of the enalapril and ramipril agents did not alter regional CBF significantly, showing their safety in hypertensive patients with CAS of high grade. This is important in minimizing the risk of cerebral hypoperfusion while effectively controlling systemic hypertension [31]. Moreover, according to Chang *et al*. (2013), ACE inhibitors decreased stroke events and cardiovascular deaths in asymptomatic CAS, consequently supporting the outcomes [27].

**Figure 3:**
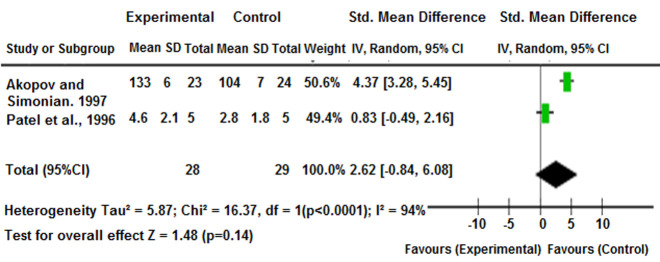
forest plot of standardized mean differences between experimental and control groups across two studies on the effect of enalapril on rCBF

For meta-analysis (Figure 3), statistical data from two included studies were used to assess the effectiveness of enalapril on regional CBF (rCBF) in elderly asymptomatic CAS patients [28,31]. The pooled analysis favored the experimental group with standardized mean differences (SMD = 2.62, 95% CI: -0.84, 6.08), though results were statistically insignificant (P = 0.14) and show high heterogeneity (I^2^= 94%).

**Calcium channel blockers (CCBs) treatment:** CCBs reduced cardiovascular events and improved vascular hemodynamics among patients with asymptomatic CAS, in which isradipine enhanced carotid hemodynamics without altering rCBF. According to Akopov and Simonian (1997), isradipine reduced systolic and diastolic BP while simultaneously enhancing blood flow in CAS [28]. The vascular resistance in the ipsilateral carotid artery was reduced through vasodilation caused by isradipine. Moreover, there were no severe cerebral perfusion impairments in patients with chronic or recent hypertension and unilateral internal CAS [28].

Additionally, lacidipine, a dihydropyridine CCB, reduced vascular remodeling and improved cerebral perfusion. Lacidipine led to a significant reduction in BP (from 165 ± 21/91 ± 8 to 154 ± 12/86 ± 12 mmHg) and carotid intima-media thickness (IMT) [29]. In addition, lacidipine protected cerebral perfusion, maintaining adequate blood flow in patients with CAS, unlike HCTZ.

Furthermore, over 36 months, amlodipine slowed the progression of carotid artery atherosclerosis [32] as in the amlodipine group, a decrease of 0.0126 mm IMT was observed (P=0.007), whereas the placebo group exhibited a 0.033 mm increase. Compared to non-CCB-based regimens, amlodipine-based regimens demonstrated a 10% reduction in the risk of overall cardiovascular events (OR: 0.90; 95% CI: 0.82-0.99; P=0.02) and total mortality (OR: 0.95; 95% CI: 0.91-0.99; P=0.01). Furthermore, amlodipine showed superior protection against stroke compared to other antihypertensive drugs (OR: 0.84; 95% CI: 0.79-0.90; P<0.00001) [32].

**Figure 4:**
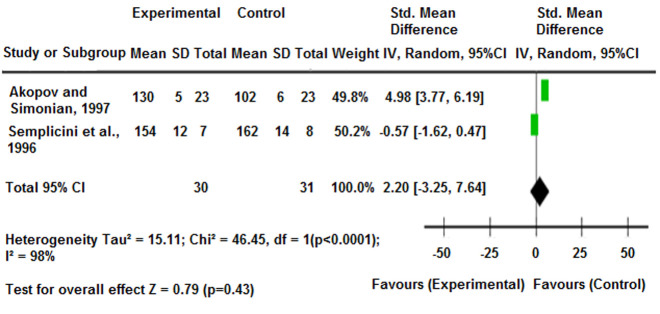
forest plot of standardized mean differences comparing experimental and control groups across two studies on the effect of CCBs on blood pressure

To assess the effect of CCBs (isradipine and lacidipine) on BP for meta-analysis, statistical data from two included studies were used (Figure 4). The study by Akopov and Simonian (1997) analyzed the effects of isradipine [28] while Semplicini *et al*. (2000) investigated lacidipine intervention [29]. The pooled estimate favored the experimental group, with an SMD of 2.20 (95% CI: -3.25 to 7.64), though the results were statistically insignificant (P=0.43).

**Thiazide diuretic treatment:** according to Zanchetti *et al*. (2004), HCTZ reduced BP (from 167 ± 16/95 ± 5 to 162 ± 24/90 ± 14 mmHg), which is crucial in mitigating the progression of atherosclerosis in asymptomatic CAS. Despite this reduction, HCTZ reported less efficacy in comparison to fosinopril for slowing the progression of carotid IMT, a key marker of atherosclerosis [30]. This highlighted that while HCTZ contributes to cardiovascular risk reduction through blood pressure management, it may not provide additional vascular protective effects beyond its antihypertensive action.

Similarly, Semplicini *et al*. (2000) compared HCTZ versus lacidipine on cerebral perfusion in a study population with hypertension and CAS. The findings revealed that HCTZ effectively reduced BP but was associated with a slight decline in CBF (from 167 ± 16/95 ± 5 to 162 ± 24/90 ± 14 mmHg), suggesting a potential risk for impaired cerebral perfusion in this patient population [29]. These findings demonstrate the need for balancing antihypertensive efficacy against the preservation of cerebral hemodynamics during the use of thiazide diuretics in patients with CAS.

**Angiotensin receptor blockers (ARBs) treatment:** ARBs reduce cardiovascular events by improving vascular health, reducing the risk of stroke, and significantly decreasing the hazard ratio for patients [27]. These agents controlled hypertension and comorbid conditions strongly associated with increased cardiovascular event risks, such as chronic kidney disease and coronary artery disease. The patients on ARB therapy reported a low incidence of stroke in comparison to the patients who were not on the same treatment, further reiterating ARBs' role in effective risk management [27]. In addition, the annual and 5-year stroke rates were 0.7% and 3.5% respectively, for ARB treatment. This shows that ARBs perform an essential function in lowering the risk of transient ischemic attacks and stroke by stabilizing BP and reducing vascular inflammation [27].

**Beta-blocker treatment:** labetalol effectively reduced mean arterial pressure (from 100 mmHg to 80 mmHg; p < 0.01) in hypertensive episodes post-carotid endarterectomy. In contrast, contralateral Vmean remained unaffected by labetalol, emphasizing the drug's targeted impact on the ipsilateral side affected by hyperperfusion [26]. In addition, metoprolol CR/XL demonstrated significant effects on plaque characteristics and cardiovascular outcomes over time. Plaques in participants treated with metoprolol for 36 months were significantly more echogenic compared with the untreated ones (57.3 ± 16.8 vs. 51.8 ± 20.0, p = 0.006, respectively) [33]. Additionally, over an 8-year follow-up, active treatment with beta-blockers reduced cardiovascular event rates by 17% versus 37% for placebo (p = 0.011), highlighting the greater benefit in individuals with pre-existing atherosclerosis [33].

## Discussion

The review investigated the impact of antihypertensive medications in asymptomatic CAS patients for reducing cardiovascular events through direct blood pressure management, plaque stabilization, and vascular remodeling. Among patients with asymptomatic CAS, significant variations were observed in the effectiveness of different antihypertensive drug categories. ACE inhibitors, particularly enalapril and fosinopril, demonstrated dual benefits by effectively lowering BP while simultaneously improving carotid hemodynamics [28,30,31]. The meta-analysis, though statistically insignificant, suggested a positive trend favoring enalapril's effect on rCBF [28,31]. CCBs like isradipine and lacidipine improved carotid hemodynamics without compromising cerebral perfusion [28,29]. Amlodipine reduced both inflammation markers and macrophage infiltration when combined with atorvastatin, leading to a 10% reduction in overall cardiovascular events [32]. However, one of the major aspects of interpreting the results from primary investigations is the impact of concomitant interventions, mainly involving statins [32]. The patient populations receiving statins along with antihypertensive medications may have contributed to improvements in cardiovascular health, as there is no isolation effect of antihypertensive intervention observed from that of concurrent lipid-lowering treatment, making blood pressure management challenging. Hence, future research should focus on independent treatments (lipid-lowering agents versus antihypertensive intervention) in enhancing cardiovascular outcomes.

Thiazide diuretics, while effective in BP reduction, showed limitations in protecting against atherosclerosis progression compared to other drug classes. HCTZ successfully reduced BP but was associated with potential risks of impaired cerebral perfusion [29,30]. ARBs demonstrated significant effectiveness in stroke prevention, with studies showing a remarkable reduction in 5-year stroke rates in treated patients.

Beta-blockers like labetalol were effective in managing post-carotid endarterectomy hypertension and cerebral hyperperfusion symptoms [26], as metoprolol CR/XL significantly improved plaque echogenicity over 36 months and demonstrated a substantial reduction in cardiovascular event rates (17% versus 37% in placebo) over an 8-year follow-up period [33]. This finding can be utilized for clinical purposes, mainly as risk stratification for asymptomatic CAS, as increased echogenicity reflects more stable plaques. Hence, beta-blockers can be used as an adjunct in stabilizing plaques along with their antihypertensive effects, as echogenicity improvements serve as a surrogate marker. The combination therapy approach, particularly with statins, showed enhanced benefits across multiple outcomes, suggesting the importance of comprehensive treatment strategies in managing CAS.

The differential effectiveness of antihypertensive medications can be attributed to their distinct mechanisms of action and their effects beyond blood pressure management. ACE inhibitors' dual benefit likely stems from their ability to modulate the renin-angiotensin system, which affects both BP and vascular remodelling [34]. This favorable effect on carotid hemodynamics without the impairment of CBF may show that such drugs execute a delicate equilibrium between systemic BP reduction and local vascular wall function.

The better performance of CCBs to maintain cerebral perfusion with a reduction in BP may be related to their direct vasodilatory effects on the cerebral vasculature [35,36]. Increased efficacy of combination therapy with statins suggests a synergistic action through both hemodynamic and inflammatory pathways in atherosclerosis [37]. The limited efficacy of thiazide diuretics in preventing the progression of atherosclerosis despite good blood pressure management would suggest that a reduction in BP alone is not sufficient for optimal vascular protection.

The benefit of ARBs on stroke rates implies that, apart from their antihypertensive properties, the stabilization of plaques and vascular inflammation reduction due to this class of drugs occurs along with the aforementioned mechanism. Beta-blockers control post-endarterectomy complications and temporal improvement of plaque characteristics. The limited efficacy of thiazide diuretics in preventing the progression of atherosclerosis, despite blood pressure management, underscores the need for targeting multiple pathways in vascular disease. The reduction in stroke rates with modern medical management, particularly with ARBs, represents a significant improvement over historical data from studies involving asymptomatic CAS, highlighting the evolution of treatment strategies. The findings regarding beta-blockers' effects on plaque characteristics provide new evidence supporting their use in specific clinical scenarios, expanding upon previous knowledge of their cardiovascular benefits.

**Strengths and limitations:** this review comprehensively evaluated multiple classes of drugs, providing a wide-ranging understanding of their effects. In addition, it presents an elaborate evaluation of several cardiovascular outcomes, offering key aspects related to the impact of antihypertensive treatments. However, the heterogeneity in the design of the studies and their various measures of outcome limits the general applicability of the results. In addition, the limited availability of a long-term follow-up might pose a challenge for assessing the sustained effect of interventions. Moreover, the review focused on and included studies published in the English language, limiting the recruitment of key information from the studies published in other languages.

Furthermore, the review highlighted the need for individualized selection of antihypertensive therapy, depending on the severity of stenosis and a wide range of patient characteristics. Treatments considered by clinicians should take into account not only BP-lowering efficacy but also plaque-stabilizing properties. The findings also emphasized the use of comprehensive cardiovascular risk assessment in treatment decisions and underscores the importance of follow-up monitoring of the progression of CAS in treated patients. Additionally, future studies should investigate cardiovascular outcomes over the long-term follow-up period, optimal targets of BP concerning the various severities of stenosis, and combination therapies. In addition, further studies should be carried out on personalized treatment approaches according to the molecular and imaging markers of plaque vulnerability.

## Conclusion

The review provides valuable insights related to the impact of antihypertensive treatments in asymptomatic CAS patients for reducing cardiovascular events. Managing cardiovascular events is complex, and different antihypertensive treatments have different mechanisms of action, with varying efficacies in stabilizing carotid plaques and reducing cardiovascular events. ACE inhibitors and CCBs demonstrate superior outcomes in providing both blood pressure management and vascular protection, though with varying levels of statistical significance. Additionally, the effectiveness of combination therapy, especially with statins, highlights the importance of multi-modal treatment approaches. Moreover, beta-blockers effectively managed post-surgical complications and improved plaque characteristics. This highlights the evolution and complexity of managing asymptomatic CAS with antihypertensive therapy. Hence, the differential effects of antihypertensive treatments on vascular health, cerebral perfusion, and long-term outcomes emphasize the significance of personalized treatment approaches.

### What is known about this topic

- Asymptomatic carotid artery stenosis (CAS) is a major risk factor for cardiovascular events, including stroke, myocardial infarction, and cardiovascular death, despite the absence of overt neurological symptoms;
- Antihypertensive treatments, including ACE inhibitors, calcium channel blockers, beta-blockers, and thiazide diuretics, play a crucial role in managing hypertension and preventing cardiovascular complications;
- The efficacy of different antihypertensive drug classes in reducing cardiovascular events and their impact on carotid plaque progression and stability remains an area of ongoing research.

### What this study adds

- This systematic review and meta-analysis provide a comparative evaluation of various antihypertensive treatments in asymptomatic CAS patients, highlighting their differential effects on vascular health, plaque stability, and cardiovascular event reduction;
- ACE inhibitors and calcium channel blockers demonstrate superior benefits in blood pressure control and vascular remodeling, while beta-blockers and ARBs show significant efficacy in stroke prevention and post-endarterectomy hypertension management;
- The study underscores the importance of individualized antihypertensive therapy based on patient-specific factors and stenosis severity, emphasizing the potential for combination treatments to optimize cardiovascular risk reduction.

## Annex

**Annex 1**: quality assessment results of the cohort studies using the Newcastle-Ottawa scale (PDF - 156KB)
